# The relationship between preoperative creatinine clearance and outcomes for patients undergoing liver transplantation: a retrospective observational study

**DOI:** 10.1186/1471-2369-14-37

**Published:** 2013-02-14

**Authors:** Urs Wenger, Christian E Oberkofler, Manuel Zimmermann, Paul A Stehberger, Marcel Scherrer, Reto A Schuepbach, Silvia R Cottini, Peter Steiger, Markus Béchir

**Affiliations:** 1 Surgical Intensive Care Medicine, University Hospital of Zurich, Raemistrasse 100, Zurich, CH 8091, Switzerland; 2 Department of Visceral- and Transplantation Surgery, University Hospital of Zurich, Zurich, Switzerland

**Keywords:** Acute renal failure, Acute on chronic renal failure, Renal replacement therapy, Liver transplantation

## Abstract

**Background:**

Renal failure with following continuous renal replacement therapy is a major clinical problem in liver transplant recipients, with reported incidences of 3% to 20%. Little is known about the significance of postoperative acute renal failure or acute-on-chronic renal failure to postoperative outcome in liver transplant recipients.

**Methods:**

In this post hoc analysis we compared the mortality rates of 135 consecutive liver transplant recipients over 6 years in our center subject to their renal baseline conditions and postoperative RRT. We classified the patients into 4 groups, according to their preoperative calculated Cockcroft formula and the incidence of postoperative renal replacement therapy. Data then were analyzed in regard to mortality rates and in addition to pre- and peritransplant risk factors.

**Results:**

There was a significant difference in ICU mortality (p=.008), hospital mortality (p=.002) and cumulative survival (p<.0001) between the groups. The highest mortality rate occurred in the group with RRT and normal baseline kidney function (20% ICU mortality, 26.6% hospital mortality and 50% cumulative 1-year mortality, respectively). The hazard ratio in this group was 9.6 (CI 3.2-28.6, p=.0001).

**Conclusion:**

This study shows that in liver transplant recipient’s acute renal failure with postoperative RRT is associated with mortality and the mortality rate is higher than in patients with acute-on-chronic renal failure and postoperative renal replacement therapy.

## Background

Renal failure with following continuous renal replacement therapy (RRT) is a major clinical problem in liver transplant recipients with reported incidences of 3% to 20% [1-3]. Apart from higher costs renal failure is associated with increased mortality in ICU patients in general [4] and in particular in liver transplant recipients, varying from 27% to 67% depending on the comorbidities [5-7].

Preoperative renal dysfunction increases the intraoperative complications and is a strong predictor of mortality [8]. Gonwa et al. reported that 35% of liver transplant recipients with hepatorenal syndrome (HRS) needed RRT postoperatively versus only 5% without HRS [9]. Although TPL can correct HRS, interestingly the renal function often recovers only to a glomerular filtration rate of 30-40 ml/min. Potential explanation seems to be the administration of immunosuppressants in these cases [10,11]. Furthermore, renal failure is leading to prolonged hospital stay, increased rate of rejection and rate of infection in liver transplant recipients [8]. There are many reports regarding preoperative renal function, especially the role of preoperative creatinine serum levels in post-operative outcomes in liver transplant recipients [12-14].

Apart from preexisting renal impairment, i.e. HRS [15], there are other stressors for the kidneys: Intraoperative occurrence of hypotension with/or without hypovolemia may reduce renal perfusion [16] and operation without veno-venous bypass may lead to renal congestion resulting in further renal injury [17]. Postoperatively used nephrotoxic agents like antibiotics or immunosuppressants may further contribute to progressive renal failure [18]. Therefore, it is difficult to distinguish between pretransplant and peritransplant factors contributing to the pathogenesis of renal failure resulting in RRT in liver transplant recipients. The calculated Cockcroft formula is easy to assess and is of clinical importance because for instance renal impairment dosages of drugs are estimated by calculating the estimated creatinine clearance using this formula. Little is known about the significance of the preoperative calculated Cockcroft formula in assessing postoperative outcome in liver transplant recipients. There are concerns about its use in the context of advanced cirrhosis or fulminant liver failure [19]. Therefore, in this post hoc analysis we compared the mortality rates of 135 consecutive liver transplant recipients over 6 years in our center subject to their renal baseline conditions and postoperative RRT. Furthermore, we tried to identify pre- and perioperative risk factors for the development of acute or acute-on-chronic renal failure after liver transplantation.

## Methods

We included 135 consecutive liver transplant recipients between January 1, 2003 and December 31, 2008 operated in our center. Following approval by the local Ethics (KEK 4 Kantonale Ethikkomission, Abt. 4) all patients gave written informed consent before transplantation for data analysis post transplantation.

### Inclusion/exclusion criteria

We included all adult (>16 years) regularly and high urgently listed liver transplant recipients between January 1, 2003 and December 31, 2008 in our transplantation center. Exclusion criteria were retransplantation, combined transplantations (e.g. liver and kidney), renal replacement therapy before TPL, preexisting kidney transplantation and living donor related liver transplant recipients.

### Pretransplant recipient data

As baseline characteristics we analysed age, gender, BMI, creatinine, creatinine clearance estimated by Cockcroft formula, haematocrit and platelet count. Normal creatinine clearance was defined as > 60 ml/min. Furthermore, the following clinical data were collected: Underlying liver disease, MELD score [20] (corrected and uncorrected) [21], Child classification of liver cirrhosis, incidence of diabetes mellitus and location directly before TPL (home, hospital or ICU).

### Operative data

All patients were transplanted without veno-venous bypass, as described by McCormack et al. [22]. Patient records were analysed in respect to ASA class, operating time, estimated intraoperative blood loss, transfusion of RBC, FFP or platelets intraoperatively and the application of Fibrinogen.

Marginal grafts were defined as either age ≥ 65 years or cold ischemia time ≥ 720 min or biopsy-proven steatosis (micro- or macrovascular in ≥ 60% of hepatocytes or ≥ 30% macrovascular steatosis) [23,24].

### ICU data

The following data were collected: Serum peak values of bilirubin, ALT and AST, length of stay in the ICU, readmission rate to the ICU, postoperative serum creatinine peak level, incidence of renal replacement therapy, incidence of sepsis defined according to the international guidelines [25], incidence of pulmonary failure (ARDS defined according to the AECC definition [26], pneumonia in need of reintubation), incidence of reoperations during ICU stay.

### Post-hoc analyzing protocol

After collection of this data we classified the patients into 4 groups, according to (1) their preoperative calculated Cockcroft formula and (2) the postoperative need of RRT. (We used the equation: (140-age)*body weight/creatinine{mg/dl}*72 for Cockcroft formula (multiplied with 0.85 in the case of women):

*Group 1:* Calculated Cockcroft formula > 60 ml/min with postoperative RRT.

*Group 2*: Calculated Cockcroft formula > 60 ml/min without postoperative RRT.

*Group 3:* Calculated Cockcroft formula < 60 ml/min with postoperative RRT.

*Group 4:* Calculated Cockcroft formula < 60 ml/min without postoperative RRT.

We calculated and compared the ICU mortality, hospital mortality and mortality overall and analysed the cumulative survival of the 4 different groups.

Then we performed univariate analysis of risk factors between the groups with normal creatinine clearance (group 1 and 2) and decreased creatinine clearance (group 3 and 4), respectively.

### Statistical analysis

The between group comparisons were made with Mann and Whitney U-test for continuous variables and Chi-tests for nominal variables. Cumulative survival analysis was done by the method of Meier Kaplan. All calculation and analysis were done with Stat view 4.5 (abacus concepts, Berkeley, CA, USA). Statistical significance was accepted with p< 0.05 (two sided tests).

## Results

### Distribution of the groups

The number of 135 patients was assigned to the 4 groups as follows: Group 1 consisted of 15 (11.1%) patients, group 2 of 85 (63.0%), group 3 of 10 (7.4%) and group 4 consisted of 25 (18.5%), respectively. Baseline characteristics and diagnoses are given in Tables 1 and 2.

**Table 1:** Baseline characteristics (n=135)

**Men/women**	**104/31**
Age (yrs.)	51.1±12.0 (18.0–70.5)
Weight (kg)	77.6±16.0 (43.0–136.0)
Height (m)	1.73±0.10 (1.50–1.95)
BMI (kg/m^2^)	25.8±4.3 (16.0–36.0)
Creatinine (μmol/l)	99±44 (40–306)
Hematocrit (%)	32.4±6.6 (19.3–49.6)
Platelets (10^3^/μl)	103±60 (22–285)

Data expressed as mean ± SD (range).

**Table 2:** Underlying liver diseases (n=135)

HCV liver cirrhosis overall	51 (37.7%)
HCV liver cirrhosis + HCC	18 (12.8%)
HBV liver cirrhosis overall	13 (9.6%)
HBV liver cirrhosis +HCC	6 (4.4%)
HCC overall	37 (27.4%)
Alcoholic liver cirrhosis overall	24 (17.7%)
Alcoholic liver cirrhosis + HCC	1 (0.7%)
PSC	5 (3.7%)
PBC	4 (2.9%)
Wilson’s disease	4 (2.9%)
Cryptogenic liver cirrhosis	2 (1.5%)
Amyloidosis	3 (2.2%)
Budd chiari syndrome	2 (1.5%)
Alpha-1-antitrypsin deficiency	1 (0.7%)
AIH liver cirrhosis	1 (0.7%)
Osler’s disease	1 (0.7%)
Other	2 (1.5%)
Acute liver failure	11 (8.1%)

*HCV* indicates hepatitis C virus, *HBV* hepatitis B virus, *HCC* hepatocellular carcinoma, *PSC* primary sclerosing cholangitis, *PBC* primary biliary cirrhosis and *AIH* autoimmune hepatitis, respectively.

### Mortality

**Figure 1:**
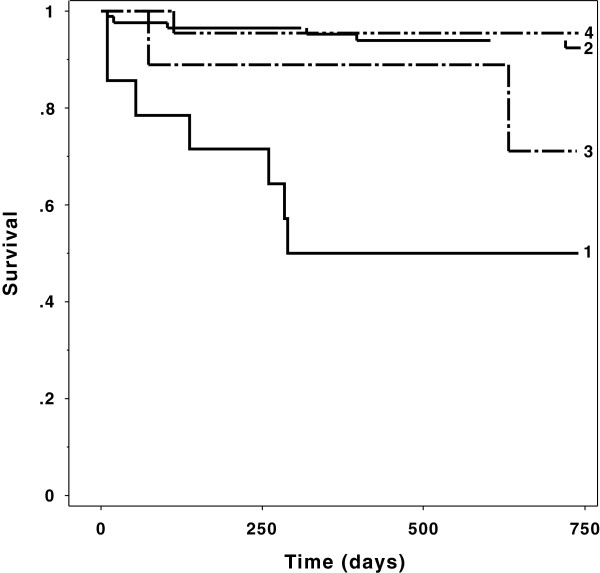
**Kaplan Meier curve of the 4 groups: There were significant different survival rates.** The lowest cumulative survival shows group 1, with preoperative normal kidney function and postoperative renal replacement therapy (p<.0001, log rank test)

There was a significant difference in ICU mortality (p=.008), hospital mortality (p=.002) and overall mortality (p=.015) between the groups. Interestingly, the highest mortality rate occurred in the group with RRT and normal baseline kidney function (20% ICU mortality, 26.6% hospital mortality and 50% cumulative 1-year survival, respectively). For details see Figure 1 and Table 3.

**Table 3:** Mortality

**Mortality**	**Group 1**	**Group 2**	**Group 3**	**Group 4**	**p-value**
ICU (%)	20.0	2.4	0	0	0.008
Hospital (%)	26.7	2.4	10.0	0	0.002
Overall (%)	46.7	12.9	20.0	8.0	0.015

Comparison with Chi-Test.

Group 1: Calculated Cockcroft formula ≥ 60 ml/min with postoperative RRT.

Group 2: Calculated Cockcroft formula ≥ 60 ml/min without postoperative RRT.

Group 3: Calculated Cockcroft formula ≤ 60 ml/min with postoperative RRT.

Group 4: Calculated Cockcroft formula ≤ 60 ml/min without postoperative RRT.

### Differences between acute renal failure with RRT and acute-on-chronic renal failure with RRT in regard on the risk of mortality

The hazard ratio in the group 1 (acute renal failure with RRT) was 9.6 (CI 3.2-28.6, p=.0001) and in the group 3 (acute-on-chronic renal failure with RRT) 5.6 (CI 0.5-62.5, p=.15).

### Which factors contributed to RRT in patients with “normal” baseline kidney function (group 1, acute renal failure)?

In 15 out of 100 patients (15.0%) with normal preoperative kidney function RRT was necessary during the ICU stay.

Univariate analysis revealed BMI (p=.05), hematocrit before TPL (p=.014) and MELD score (p=.014) as preoperative risk factors for RRT after transplantation. Intraoperatively, the estimated blood loss (p=.05) was a risk factor for RRT and there was a trend towards fibrinogen application (p=.08). Furthermore, there were on one hand significant different AST (p=.015) and ALT (p=.022) peak levels and on the other hand significant increased bilirubin (p=.003) and alkaline phosphatase (p=.002) peak serum levels in the group with RRT (group 2) versus the group without RRT (group 2).

In the ICU the patients requiring RRT postoperatively (group 1) had significant more sepsis (p=.0001) and respiratory failure with the need for reintubation (p=.009); had more readmissions to the ICU (p=.006) and there was a significant higher reoperation rate (p=.0014). Furthermore, the length of stay in the ICU was also dramatically increased in this patient group (p=.0003).

In contrast there were no differences between the groups in respect to gender, age at TPL, platelet count before TPL, Child stadium, incidence of diabetes mellitus, and location before TPL (hospital/ICU/home), the rate of marginal donor grafts, operating time, and transfusion of RBC, FFP or platelets. For details see Table 4.

**Table 4:** Patients with normal baseline creatinine clearance (> 60 ml/min)

	**Group 1 (n=15)**	**Group 2 (n=85)**	**p–value**
Gender (m/f)	2/13	24/61	0.22
Age (yrs.)	58 (30–65)	52 (16–69)	0.39
BMI (kg/m2)	26.7 (23.2–36.0)	24.8 (16.0–36.2)	0.05
Hematocrit before TPL (%)	31.5 (15.3–40.0)	35.3 (21.7–49.6)	0.01
Platelets before TPL (10^3^/μl)	83 (42–185)	92 (27–285)	0.32
MELD (corrected)	23 (6–31)	17 (7–33)	0.014
MELD (uncorrected)	21 (6–31)	12 (6–32)	0.13
CHILD (A/B/C in %)	23.1/30.8/46.1	35.4/41.8/22.8	0.20
Diabetes mellitus (%)	33.3%	16.8%	0.12
Admission from (Home/ward/ICU in %)	66.6/13.3/20.0	88.2/4.7/7.1	0.09
ASA class (II/III/IV in %)	6.7/53.3/40.0	11.8/61.2/27.0	0.55
Operating time (min)	390 (275–705)	355 (240–570)	0.11
Estimated intraoperative Blood loss (ml)	3000 (500–15000)	1000 (300–10000)	0.05
Marginal grafts (%)	46.7	35.3	0.40
Transfusion of – RBC (Unit)	2 (0–47)	3 (0–23)	0.36
- FFP (Unit)	12 (0–77)	12 (0–50)	0.62
- Platelets (Units)	1 (0–12)	0 (0–18)	0.37
- Fibrinogen (g)	2 (0–22)	0 (0–12)	0.08
Length of stay in ICU (days)	8 (3–54)	4 (2–31)	0.0003
Readmissions (%)	33.3	16.5	0.006
Incidence of Sepsis (%)	40.0	3.5	0.0001
Incidence of Respiratory Failure (w. reintubation) (%)	26.7	5.9	0.009
Reoperations (%)	40.0	10.6	0.001
Bilirubin peak (μmol/l)	157 (65–475)	87 (13–453)	0.003
ALT peak (U/l)	1625 (346–5147)	870 (133–7249)	0.02
AST peak (U/l)	1926 (389–10740)	1047 (114–13560)	0.02

Data expressed as median (range) or number of patients (percentage), *TPL* indicates transplantation, *MELD* model for end stage liver disease and *ICU* intensive care unit, respectively.

### Renal failure in the group with normal baseline condition and without postoperative RRT (group 2)

In the group 1 with RRT the mean peak creatinine level postoperative before starting RRT was 260±98 μmol/l, whereas in the group 2 without RRT the peak level was 136±68 μmol/l indicating that even patients without RRT developed renal failure after TPL.

### Which factors contributed to RRT in patients with preexisting impaired kidney function before TPL (group 3, acute-on-chronic renal failure)?

In 10 of 32 patients (31.3%) with impaired baseline creatinine clearance postoperatively RRT was necessary, i.e. in 22 patients (68.7%) no RRT had to be used.

Univariate analysis displayed BMI (p=.028) as preoperative risk factor and transfusion of RBC (p=.016) and fibrinogen (p=.039) as intraoperative risk factors for RRT after TPL. As in the group with normal baseline kidney function in the ICU the patients requiring RRT postoperatively (group 3) also had significant more sepsis (p=.021) and respiratory failure with reintubation (p=.039); had more readmissions back to the ICU (p=.031), showed a significant higher reoperation rate (p=.036) and also the length of stay in the ICU was dramatically increased in this patient group (p=.0002).

The other analyzed factors did not differ between the 2 groups. For details see Table 5.

**Table 5:** Patients with impaired baseline creatinine clearance (< 60 ml/min)

	**Group 3 (n=10)**	**Group 4 (n=25)**	**p-value**
Gender (m/f)	10/0	20/5	0.12
Age (yrs.)	56 (36–68)	49 (23–70)	0.18
BMI (kg/m2)	26.7 (23.2–31.8)	23.7 (18.7–33.0)	0.03
Hematocrit before TPL (%)	26.8 (18.4–38.3)	28.2 (21.6–40.7)	0.56
Platelets before TPL (10^3^/μl)	51 (40–158)	83 (22–253)	0.97
MELD (corrected)	22 (19–33)	23 (11–36)	0.55
MELD (uncorrected)	21 (19–28)	16 (6–36)	0.19
CHILD (A/B/C in %)	0/0/100	15/20/65	0.15
Diabetes mellitus (%)	10.0%	8.0%	0.85
Admission from (Home/ward/ICU in %)	30.0/40.0/30.0	60.0/28.0/12.0	0.23
ASA class (II/III/IV in %)	0/30.0/70.0	8.0/28.0/64.0	0.65
Operating time (min)	362 (250–660)	367 (260–480)	0.50
Estimated intraoperative Blood loss (ml)	3000 (200–10000)	1500 (500–8000)	0.31
Marginal grafts (%)	60	36	0.19
Transfusion of - RBC (Unit)	13 (2–39)	7 (0–20)	0.02
- FFP (Unit)	10 (0–55)	14 (0–36)	0.85
- Platelets (Units)	3 (0–6)	1 (0–12)	0.22
- Fibrinogen (g)	7 (0–22)	2 (0–20)	0.04
Length of stay in ICU (days)	10 (5–94)	4 (2–18)	0.0002
Readmissions (%)	60.0	16.0	0.03
Incidence of Sepsis (%)	20.0	0	0.02
Incidence of Respiratory Failure (w. reintubation) (%)	30.0	4.0	0.04
Reoperations (%)	50.0	12.0	0.04
Bilirubin peak (μmol/l)	128 (57–535)	119(14–568)	0.42
ALT peak (U/l)	656 (133–8492)	632 (95–4727)	0.91
AST peak (U/l)	922 (183–13805)	979 (119–4305)	0.68

Data expressed as median (range) or number of patients (percentage), *TPL* indicates transplantation, *MELD* model for end stage liver disease and *ICU* intensive care unit, respectively.

## Discussion

As main finding this study revealed the highest mortality rate in the group of liver transplant recipients with normal preoperative kidney function and postoperative renal replacement therapy.

The main limitation of this study is the sample size of only 135 patients leading to small analyzing groups (e.g. n=15 in group 1). Therefore, the interpretation of this data must be done very carefully. The use of Cockcroft formula as stratifying tool has its limitation, as there is evidence that proteinuria [27] or MDRD [28] might perform better in pointing out renal pathology or postoperative outcome. Unfortunately, in our retrospective patient collective there was no routinely testing of proteinuria. Thus, we cannot give information about the comparison and accuracy of proteinuria and Cockcroft formula. Actually we took Cockcroft formula because it is the easiest and most historical and best known and used estimation for renal function and nevertheless, its prediction of postoperative outcome in liver transplanted recipients is of clinical importance.

The group with acute renal failure and RRT showed also higher MELD scores and lower pretransplant hematocrit values than the patients without postoperative RRT and normal baseline kidney function. This might indicate higher severity of baseline liver disease in these patients. It is noteworthy that Weismüller et al. reported increased postoperative creatinine levels in 56 patients with MELD >16 versus a group with MELD <16, indicating an influence of MELD score on postoperative renal failure [29], but in contrast Faenza et al. could not find an influence of MELD on postoperative renal failure [7]. Potential explanation for the highest mortality might be more postoperative liver cell damage or impaired function – indicated by elevated liver enzymes and bilirubin peak serum levels- with consecutive multiorgan-dysfunction-syndrome, inclusive renal dysfunction, infections and finally with mortality as shown in Tables 3 and 4. Therefore, the use of marginal grafts might contribute to such a postoperative course as described previously [30]. Our data favors the concept that MELD score and in turn severity of liver disease correlates with postoperative renal failure and RRT.

Intraoperative transfusion of RBC was associated with an increased incidence of RRT and mortality in the group with preexisting renal failure, but not in the group with normal renal baseline conditions.

There is strong evidence that transfusion of RBC can compromise critically ill patients [31-33] and there are reports, which identified intraoperative transfusion as a risk factor for morbidity, i.e. renal failure and mortality in liver transplant recipients [16,34,35]. On that basis, the strategy to transfuse as few RBC intraoperatively as possible seems to be a promising concept and a high priority aim [36,37]. More blood loss intraoperatively might lead to reduced survival in liver TPL [38]. Interestingly, our study group 3 had also significant more intraoperative blood loss, which is in line with that data. After the study by Massicotte et al. low CVP and restrictive transfusion regime were strongly propagated [37], but the excellent results in that study occurred in a study population with mean MELD of 18. Whether this strategy is also as beneficial in sicker patients with higher MELD scores, e.g. 21 in our study remains still to be determined. During liver transplantation and its long and difficult anesthesia the patients might become hypovolemic and hypotensive resulting in impaired organ perfusion, i.e. kidney perfusion and in turn might contribute to postoperative renal failure [39]. Furthermore, the reperfusion after portal unclamping results in decreased heart rate, contractility and peripheral vascular resistance leading also to cardiovascular instability [40]. Therefore, the concept of “keep the patient dry” must be examined very carefully, in particularly in regard to the data reported by Schroeder et al., which reported more renal failure and RRT with worse survival in a low CVP group [41].

The two RRT groups (1 and 3) received also significant more fibrinogen transfused. This might contribute to renal failure because of micro embolic effects with clotting appearing in the capillaries of the kidney, there is data concerning thromboembolic events after administration of fibrinogen [42].

Taken together, impaired kidneys seem to be more vulnerable to transfusion of RBC and fibrinogen than normal kidneys. Potential explanation might be that a minimal amount of clotting in preexisting impaired renal parenchyma and vasculature provokes a significant additional organ damage resulting in renal injury.

Significant increased postoperative peak levels of ALT and AST indicating severe hepatocellular damage were associated with higher incidence of RRT in patients with normal baseline kidney function. This finding might be due to toxic or inflammatory effects of mediators released by ischemic liver cells. Another explanation might be hypoperfusion with impaired microcirculation in both the liver graft and the kidneys during postoperative SIRS or the use of marginal grafts, which is reported to increase morbidity [43]. However, in this study we could not show a correlation between marginal grafts and mortality.

Our data demonstrate that the complete loss of renal function (acute renal failure) with RRT is a strong predictor of death and in turn determines outcome of liver transplanted patients. In contrast, interestingly loss of an “incomplete” renal function (acute-on-chronic renal failure) seems to be of minor importance in terms to mortality and outcome.

The fact that in 2 thirds of the patients with preexisting renal function no RRT is necessary, gives evidence that cautious operative strategy and prudent perioperative management can avoid postoperative RRT. Beneficial strategies might be intraoperatively as few transfusion of RBC as possible [36], avoiding hypotension or hypovolemia [16], maybe veno-venous bypass [17,44] or piggy back technique [45]. Postoperatively one should withhold nephrotoxic medication if possible. There are immunosuppressant protocols including sirolimus or mycofenolate mofetil-based regimes [46,47].

## Conclusions

This study shows that in liver transplant recipients acute renal failure with postoperative RRT is significantly associated with mortality and the mortality rate is higher than in the case of acute-on-chronic renal failure with RRT. Furthermore, transfusion of RBC increases the risk of RRT and mortality in acute-on-chronic renal failure.

## Abbreviations

RRT: Renal replacement therapy; HRS: Hepato renal syndrome; TPL: Transplantation; BMI: Body mass index; MELD: Model of end stage liver disease; RBC: Red blood cells; FFP: Fresh frozen plasma; CVP: Central venous pressure; AST: Aspartate aminotransferase; ALT: Alanine aminotransferase; ICU: Intensive care unit; ASA: American Society of Anesthesiologists; ARDS: Acute respiratory distress syndrome; AECC: American-European Consensus Conference; SIRS: Systemic inflammatory response syndrome; MDRD: Modification of diet in renal disease.

## Competing interests

The authors declare that they have no competing interests.

## Authors’ contributions

CEO, MZ and SRC collected the majority of the data and drafted parts of the manuscript. MS, RAS and TAN performed statistical analysis. PS and PAS helped analysing and interpreting the data and drafted parts of the manuscript. UW and MB led the project, collected parts of the data, performed additional statistical analysis and drafted parts of the manuscript. All authors read and approved the final manuscript.

## Pre-publication history

The pre-publication history for this paper can be accessed here:

http://www.biomedcentral.com/1471-2369/14/37/prepub
